# Usable Pasts Forum: UNESCO and Heritage Tourism in Africa

**DOI:** 10.1007/s10437-021-09454-6

**Published:** 2021-08-20

**Authors:** Shadreck Chirikure, Webber Ndoro, Flordeliz T. Bugarin, Savino di Lernia, Elgidius B. Ichumbaki, Noel B. Lwoga

**Affiliations:** 1 School of Archaeology, University of Oxford, Oxford, UK; 2 Department of Archaeology, University of Cape Town, Cape Town, South Africa; 3 Department of Archaeology, University of Cape Town, Cape Town, South Africa; 4 African Studies Department, Howard University, Washington, DC, USA; 5 Dipartimento di Scienze dell’Antichità, Sapienza University of Rome, Rome, Italy; 6 School of Geography, Archaeology, and Environmental Studies, University of Witwatersrand, Johannesburg, South Africa; 7 Department of Archaeology and Heritage Studies, University of Dar Es Salaam, Dar es Salaam, Tanzania; 8 National Museum of Tanzania, Dar es Salaam, Tanzania

## UNESCO and Heritage Tourism in Africa

Shadreck Chirikure

### Introduction

As an established inter-state organization, UNESCO continues to play an increasingly powerful role in the identification, conservation, and consumption of World Heritage sites across the globe, including Africa. World Heritage sites are designated by UNESCO according to the 1972 *Convention Concerning the Protection of the World’s Cultural and Natural Heritage*. UNESCO World Heritage sites appear in three major categories—cultural, natural, and a mix of both—all of which are deemed to possess Outstanding Universal Value and, consequently, are unique assets for humankind (Ndoro, 2015a). Nominated sites must, after deliberations and input from advisory bodies to the UNESCO World Heritage Committee, meet at least one of the ten criteria (six cultural and four natural) consonant with cultural, historical, scientific, or other forms of significance (Galla, 2012).

**Fig. 1:**
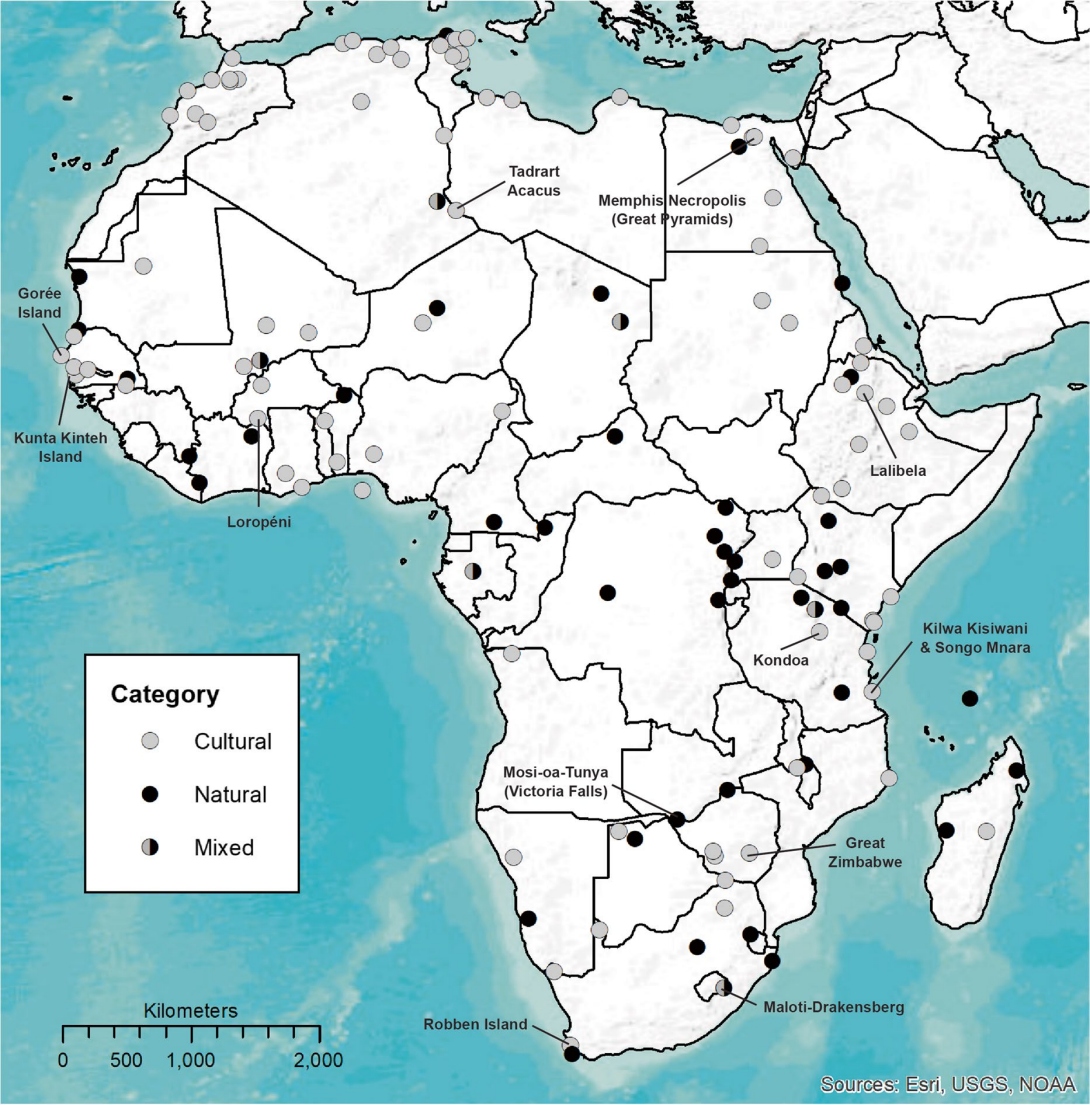
Map of UNESCO World Heritage sites in Africa (Image C. Gokee)

As of March 2021, Africa has 145 out of a total of 1,121 sites on the UNESCO World Heritage List (Fig. 1). South Africa (10) currently has the highest number, closely trailed by Ethiopia and Morocco (9), Tunisia (8), and Algeria, Egypt, Kenya, Senegal, and Tanzania (7). Some African countries have as few as one, while others have none. In contrast, China and Italy have a whopping 55 sites each, followed by Spain (48), Germany (47), France (45), and India (38), among others with numerous declared sites. In response to this disparity, there are genuine calls by African States Parties to the 1972 Convention to increase the number of sites on the continent (Ndoro, 2015a; di Lernia, this forum).

Given the vast size of Africa, and the regionally variegated infrastructures produced through colonial histories and economies, some African sites are far from regional economic nodes, while others are within their close proximity. The Egyptian Pyramids, the Serengeti, Victoria Falls/Mosi-oa-Tunya, and Robben Island are fundamental lubricants in the global tourism wheel, while others such as the Maluti-Drakensburg region of Lesotho are cogs in the same. The reality is that the form, intensity, and flow of tourism at UNESCO World Heritage sites vary from context to context. While most sites depend more on international tourists than domestic ones, some attract neither. Consequently, having sites listed on the UNESCO World Heritage List confers benefits other than tourism and these may include fame and prestige (Ndoro, this forum).

### UNESCO and Heritage Tourism in Africa: What Are Some of the Issues?

Despite being underrepresented on the World Heritage List and having historically been denied agency by colonialism, Africa has its own heritage properties with exceptional value. The big questions, however, are: (1) Are there any tangible tourism benefits associated with World Heritage listing?; (2) Who are the major beneficiaries of tourism at World Heritage properties?; (3) Is the inequality created by labeling some heritage as World Heritage and therefore more important than others beneficial at all, especially for the “non-World Heritage” sites?; (4) Do communities have a voice and decision-making stake in World Heritage affairs?; and (5) What can be done to improve local community benefits from World Heritage-designated sites? The contributions to this forum offer a nuanced engagement with these and related issues. As seasoned scholars, residents on the continent and abroad, the contributors apply first-hand and empirical insights gained at Kunta Kinteh Islands and Related Sites (Bugarin), Kilwa Kisiwani (Ichumbaki & Lwoga), and Tadrart Acacus (di Lernia). Ndoro offers a rich continental overview, penetrating deeply into issues that resonate with other contributors. Collectively, the suggestions proffered by all the contributors have the potential to filter into policy, practice, and the way in which World Heritage is experienced or not experienced through tourism in Africa.

### Who Benefits from Visitors to UNESCO World Heritage Sites?

**Fig. 2:**
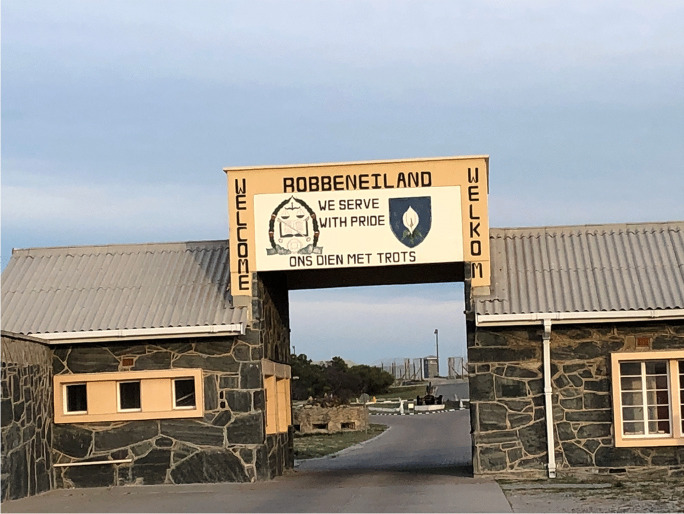
Robben Island in South Africa (Photo S. Chirikure)

UNESCO World Heritage sites are vital to different societies both in and outside Africa (Thiaw & Wait, 2018; Timothy & Nyaupane, 2009). They are integral to the present and future generations (Boswell & O'Kane, 2011). World Heritage sites are fascinating and breathtaking places to visit, but legacies of colonialism, especially those associated with infrastructure development, still condition who benefits from the associated tourism to a certain extent. Sometimes the national and international popularity of sites listed as World Heritage has less to do with how spectacular and significant they are (Bugarin, this forum; Ndoro, this forum) and more to do with how integrated they are into national and international tourism infrastructure nodes (Anderson, 2012). While some World Heritage sites are easily accessible because they had infrastructure built around them during the colonial period (e.g., Great Zimbabwe, Victoria Falls), others are located in areas where colonial infrastructures barely penetrated. Mapungubwe, which is situated in a very remote area, benefited from military infrastructure and technologies of surveillance. Robben Island is located a few nautical miles off Cape Town, one of the most popular tourism destinations in Africa (Fig. 2). In both cases, the colonial infrastructure was simply converted into facilities for tourism, but not all places have that ironic fortune. The Maloti-Drakensberg area of Lesotho was far from the colonial center, so it lacks adequate infrastructure for tourism and heritage conservation (Duval & Smith, 2013).

**Fig. 3:**
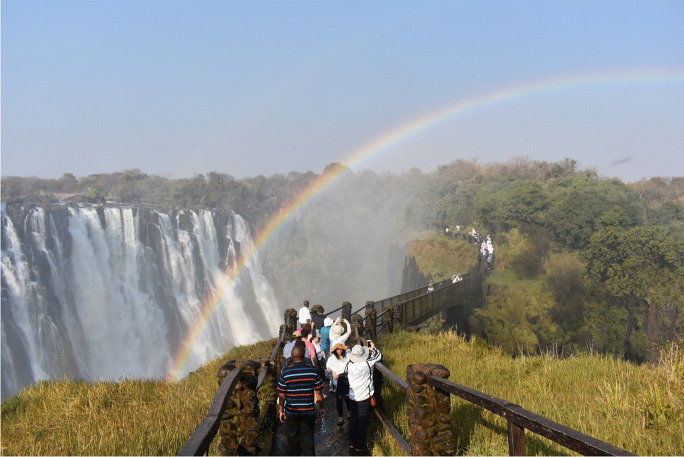
Mosi-oa-Tunya/Victoria Falls in Zambia and Zimbabwe (photo courtesy of the African World Heritage Fund)

Although inscription on the World Heritage List ensures protection from illegal activities and conditions of social conflict (di Lernia, this forum), this status does not always come with grants for conservation, and some sites have been placed on the World Heritage List in Danger (Ndoro, 2015a). Often, there is not enough tourism revenue to be reinvested in conservation. There may be validity to claims that seeking World Heritage status has, for some sites, more to do with prestige for governments than with other forms of benefit (Ndoro, 2015a; Ogundiran, 2014, 2016). Of course, there are exceptions: Kunta Kinteh, Gorée Island, the Egyptian Pyramids, Mozambique Island, and similar places all attract considerable tourism (Magnani, 2014; Thiaw & Wait, 2018; Bugarin, this forum). Also, in places such as Victoria Falls (Fig. 3), large hospitality industries and supporting infrastructures have developed in Zambia and Zimbabwe. As such, some of the best hotels in the SADC region are found at Victoria Falls. However, these are beyond the means of most domestic tourists insofar as they were constructed to tap into the tourism market for international elites.

**Fig. 4:**
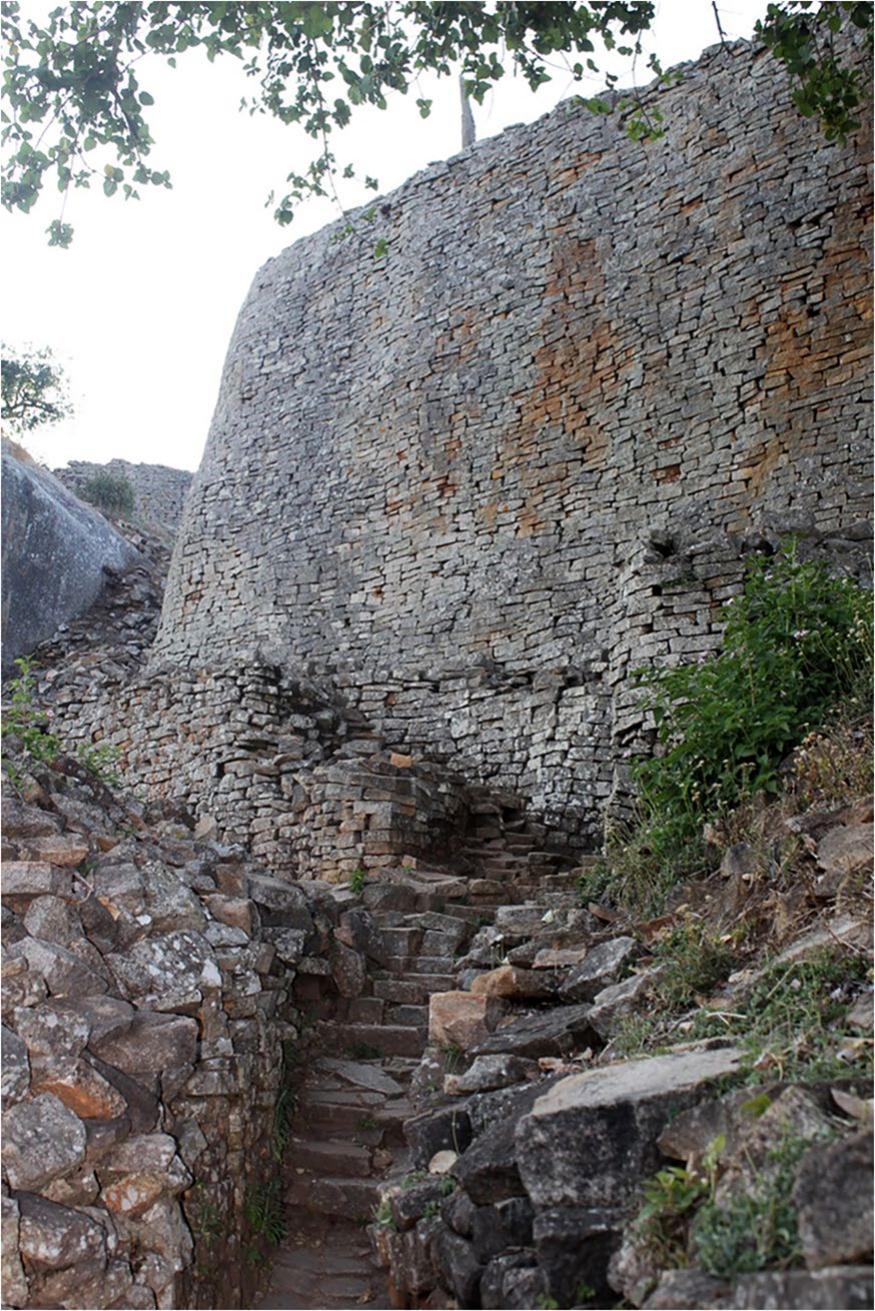
Great Zimbabwe National Monument in Zimbabwe (photo by S. Chirikure)

As Ndoro (this forum) shows, countries that had sizeable settler populations during the colonial period (e.g., Kenya, South Africa, Zimbabwe) now have a relatively well-established infrastructure for international tourists. Domestic tourism nevertheless remains underdeveloped. Even in South Africa, domestic travel can be very expensive, stunting the growth of local and regional tourism. This makes tourism vulnerable to shocks such as the COVID-19 pandemic. The decline in international tourists occasioned by the land reform and associated events in Zimbabwe circa the year 2000 resulted in the growth of local visitors to places such as Great Zimbabwe (Fig. 4). Between 2017 and 2019, the World Heritage site of Great Zimbabwe generated 300,000–400,000 USD per year, largely through domestic tourism. Perhaps lessons from COVID-19 and reduced international visitor traffic might be harnessed to better develop domestic heritage tourism in Africa. This is fundamental for instilling a sense of African pride in the past and in local resources (Kusimba, 2016; Ogundiran, 2016). The neo-colonial position that Africa requires external aid, external tourists, and the like to survive must be challenged (Chirikure, 2020; Chirikure et al., 2016).

World Heritage sites such as Kilwa Kisiwani, Lalibela, and Olduvai Gorge have also attracted academic tourists who bring in revenue during research trips. However, this tends to be episodic and often there is little money invested for conservation and there are few benefits for local communities (Ichumbaki & Lwoga, this forum). Furthermore, some of these visitors are Ph.D. students who often go on to flourishing careers, while they extract archaeological materials and export them to better-resourced laboratories abroad. There is a need to build capacity in research and conservation and to increase the number of Africans working on these sites (Kusimba, 2016; Ogundiran, 2016). Building on the positive legacy of the SIDA-SAREC initiatives led by Paul Sinclair, Gilbert Pwiti, Felix Chami, and others, increased efforts are now being made in different contexts by colleagues such as Ibrahima Thiaw, Akin Ogundiran, Sada Mire, Anne Haour, Freda Nkirote, Ann Stahl, Kristina Douglass, Innocent Pikirayi, Chap Kusimba, Webber Ndoro, Augustin Holl, and Adria LaViolette, among others, to promote inter-country research and capacity-building across borders imposed by the Berlin Conference. Some research groups (e.g., African Archaeological Network, African Framing Network) and institutions (e.g., British Institute in Eastern Africa and parallel organizations) are also contributing towards similar goals of developing sustainable cross-border networks to build capacity and train African students and early-career researchers in fieldwork, publishing, and post-excavation analyses. Hopefully, this will break the refractory neo-colonial condition in which Angolans cannot work on Zimbabwean sites, and Kenyans cannot work in Senegal, and the Senegalese cannot work in Nigeria, while Nigerians cannot work in Egypt (for similar debates, see Esterhuysen et al., 2016; Kusimba, 2016; Ogundiran, 2016; Schmidt & Pikirayi, 2018). Meanwhile, colleagues from Europe, North America, and China can chose where they want to do research, even if it means covering the entire continent.

Tourism narratives developed by Africans in Africa, ignoring the borders created by the Berlin Conference, have the potential to be transformative (Chirikure, 2020; Esterhuysen et al., 2016; Ogundiran, 2020). This is especially vital given that the African Union (AU) has come up with Agenda 2063—*The Africa We Want*—an ambitious 50-year continental development plan that pivots on cultural heritage. Building on this, the Office of the UN Under-Secretary-General and Special Adviser on Africa hosted Africa Dialogue Series panel discussions on May 26–28, 2021. The discussions internationalized the African Union’s call to use African heritage to transform mindsets, create economic opportunities, and build sustainable communities within the UN system. The hope is that tourism ventures around iconic places such as World Heritage sites may flourish into nodes for regional and community benefit (see Chirikure & Pwiti, 2008; Chirikure et al., 2010; Duval & Smith, 2013). This is an opportunity for more Africans to take initiative and engage with their continent in ways that jettison the neo-colonialism inherent in national boundaries and express their voice in narratives about Africa. Traditionally, archaeology has marginalized Africans and African communities in knowledge production, making them bystanders in a game played mostly by people from elsewhere and under rules imposed from outside.

More research by Africans will hopefully generate new narratives with the potential to empower locals to own and tell their stories and monetize them for economic benefit. Some nuance, however, is required: change will not be achieved if African scholars simply mimic existing templates and scholarly traditions that privilege Western/Eastern thought or place their work in the matrix of local narratives that simply perpetuate the status quo (Chirikure, 2020; Ogundiran, 2020). This will require revising concepts and challenging old knowledge to infuse it with new meanings produced through the full participation and meaningful engagement of local communities (Chirikure et al., 2017). Attempts can also be made to seek community validation of some narratives (Ogundiran, 2016; Schmidt & Pikirayi, 2018). This humanistic and engaged approach will transfer power, or at least share it, with African communities who can be content producers and presenters of their own narratives to tourists in a heritage agenda useful to them.

### Conclusion

World Heritage sites play a significant role in promoting tourism in Africa, though with variable results. Continual engagement and the cross-fertilization of African ideas and developments will be required to erase legacies of colonialism, and the neo-colonial convictions that continue to forestall intra-African tourism and the rise of domestic tourism in individual countries. Fulfilling such an aspiration requires multi-pronged and collaborative practices such as promoting African languages in the study of archaeology, culture, and heritage, and promoting movement across the continent by removing visa obstacles (Ogundiran, 2016). The recently formed African Continent Free Trade Area championed by the AU is a great platform to build on, in the hopes that intra-African travel becomes more affordable. However, free movement must be backed up by the construction of intellectual bridges across nations and disciplines, from the humanities and social sciences to the hard sciences, to generate holistic and integrated knowledge with potential to forge a common vision for scholars and stakeholder communities alike. This will crystallize into an African awareness fit for achieving aspirations of contemporary times. Continued collaborations and partnerships with other regions in the East and West will always be welcome but in ways that are mutually beneficial, rather than patronizing. The careerism driving some among the old and new generation of archaeologists from the West/East must be tempered with transformative and locally empowering scholarly practices at World Heritage and non-World Heritage sites on the continent.

### Acknowledgements

This piece benefited from the generous and constructive feedback, as well as the editorial guidance, of AAR editors Akin Ogundiran and Cameron Gokee, who shared ideas and suggested sources that widened my intellectual horizons. I am also indebted to my colleagues and fellow forum contributors whose thought-provoking insights and shared experiences have made it easier to draw common threads and inspire hope for archaeological practices that are transformative and beneficial to all, especially the previously marginalized.

## Cultural World Heritage Places in Sub-Saharan Africa

Webber Ndoro

### Introduction

**Fig. 5:**
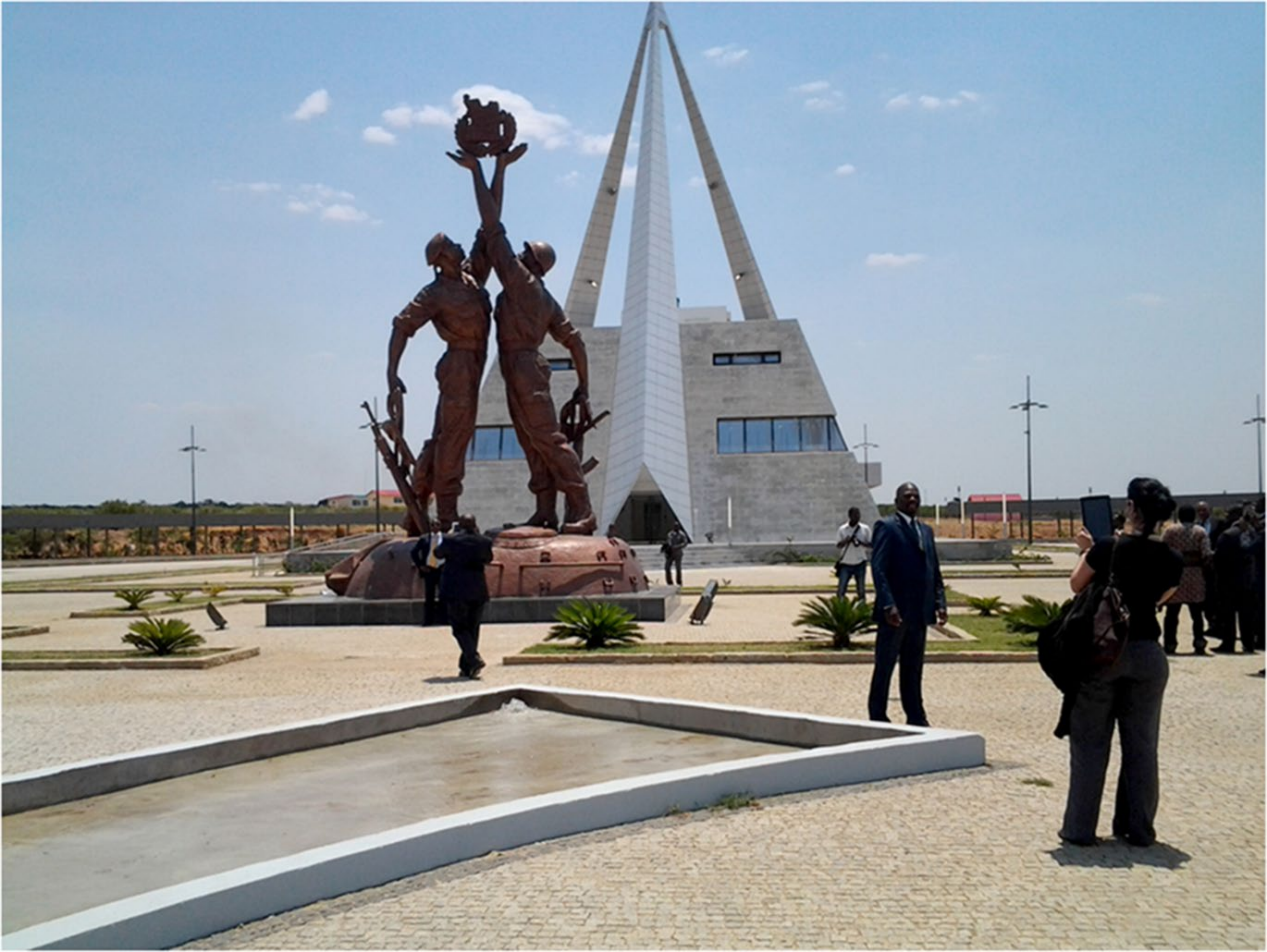
Cuito Cuanavale Memorial in Angola (photo by W. Ndoro)

In July 2020, Somalia became the fifty-fourth African country to sign the 1972 World Heritage Convention, following the signature of Southern Sudan in 2016. Thus, all countries in the African Union (except the Sahrawi Arab Democratic Republic) have signed the World Heritage Convention. African countries host a total of 145 World Heritage-designated sites, including more than 96 from Sub-Saharan countries. Most of the sites in North Africa, apart from several in Egypt and Morocco, are of Greco-Roman architecture. Most of the World Heritage sites listed for Sub-Saharan Africa are either archaeological sites (e.g., Cradle of Humankind in South Africa) or Western European architecture (e.g., Grand Bassam in Ivory Coast). Meanwhile, sites related to the genocide in Rwanda and civil war in Angola (e.g., Cuito Cuanavale [Fig. 5]) have constantly been rejected. Even Robben Island (Fig. 3), a site known throughout the world as a symbol of resistance to apartheid and colonialism, was only recognized on the World Heritage List due to what heritage experts perceive as its multi-layered history from its first use as a port where ships could stock, to a political prison in the 1700s, to a leper colony and mental hospital, and then to a WWII fort.

Across most of Africa, it was colonial archaeologists and antiquarians who initially lobbied for legislation and policies to protect heritage sites, so it is no surprise that more than 70% of those on the World Heritage List are either archaeological sites or places of colonial heritage. These two categories do not, however, adequately describe the totality of African heritage (Ndoro, 2015b).

The origins of heritage as archaeology or colonial architecture have also shaped the evolution of cultural heritage management in Africa. In general, heritage sites have been conceived as scientific specimens to be turned into protected areas. Community engagement has been limited, and in many cases, people have been moved away from heritage sites well before their nomination to the World Heritage List (Ndoro, 2001). This has less to do with the World Heritage listing than with the practice of archaeology and architectural conservation in Africa where communities are considered a danger to science (Andrews & Buggey, 2008; Ndobochani, 2016). In other words, heritage conservation and management focuses on the physical remains, expressions, and cosmologies of past societies rather than those of contemporary communities.

So, the question is what African states hope to gain from signing the World Heritage Convention? What are the intended advantages of this Convention to the member states, to the communities, and to the heritage professionals and organizations in Africa?

### The Political Thrust

For many newly independent African countries in the early 1970s, the idea of acceding to any UNESCO convention was a matter of political recognition. So, signing the World Heritage Convention had little to do with ideas of heritage; it was about nationhood and international presence. As Africans, we were taught that a nation must have a flag, a national anthem, a museum, and a heritage organization to belong to the international community. Most African government officials have struggled to define these political symbols for states where multi-ethnic cultural traits may not necessarily fit into a common national identity for all their peoples. Thus, joining the World Heritage Convention has political mileage for governments or countries, such as Somalia and Southern Sudan, clamoring for quick returns of international recognition.

### Benefits of World Heritage Status?

Acceding to the World Heritage Convention may not often be driven by conservation considerations, but the nomination of specific sites for World Heritage listing might benefit heritage in Africa. This process ensures that sites are documented and that nominating states implement better conservation and cultural heritage management plans. The nomination process also creates opportunities for the participation of different stakeholders, including local communities, in ways that deviate from the top-down policymaking and implementation in many countries. For example, the nomination of Ruins of Loropéni in Burkina Faso led to their proper 3-D documentation. And by the time the nomination process was complete, the recognition of this outstanding site had grown from less than ten Burkinabe archaeologists to include many more people across the country and around the world. The nomination process ensures that the World Heritage Committee keeps an eye on what national governments and heritage professionals are doing in terms of conservation and promotion, which for many African countries also involves the training of young people who can continue these heritage practices. An example of this process is Kondoa in Tanzania where the AFRICA 2009 program, led by UNESCO, trained professionals and stakeholders to successfully nominate the site. The AFRICA 2009 program has now trained more than 100 young professionals in Africa and resulted in the nomination of 22 World Heritage sites (UNESCO, 2010 Report).

Some believe it is elitist to focus on iconic sites for World Heritage listing in Africa, but the idea that all sites are the same only exists in the minds of archaeologists and academics. To think that Great Zimbabwe would attract the same attention as a Stone Age site (e.g., scatter of hand axes) in the same country is unrealistic.

### Cultural Heritage and Tourism in Africa

Tourism has proven to be a driver of economic growth and a source of private sector investment in some parts of Africa (Galla, 2012; Ndoro, 2015a). In South Africa, for example, international tourism arrivals and receipts grew consistently at more than 8% between 2000 and 2005, while tourism grew from 11% of total investment in 2007 to over 15% in 2019. Tourism now employs about 4 million people in Sub‐Saharan Africa. Evidence from various countries in the world, including South Africa, demonstrates that World Heritage status can serve as a local catalyst—not only for conservation, partnerships, social cohesion, skills development, and education, but also for job creation, infrastructure development, and direct investment, all leading to increases in GDP (see Galla, 2012).

In Africa, tourism is largely marketed to Western tourists, with little effort made to attract domestic tourists. Despite the changing policies of countries such as Zimbabwe, the number of tourists from Asia also remains small (Karambakuwa et al., 2011). Except for Egypt promoting the Great Pyramids, most countries attracting international tourism, including South Africa, Kenya, Botswana, and Zimbabwe, emphasize natural landscapes over cultural heritage. South Africa, for example, boasts no fewer than four World Heritage cultural sites. However, the management and policy documents of South African National Parks clearly state their mission is to promote nature-based tourism (SanParks, 2019/2020). As a result of these policies, the number of tourists visiting cultural sites in Africa is small, and the benefits are not necessarily significant for any individual country. This can only change if countries in Sub-Saharan Africa move cultural sites to the center of their tourism policy. Given the current COVID-19 pandemic, there is a need for Africa, and indeed the world, to reevaluate the marketing and promotion of world heritage sites for purposes of tourism.

### Conclusions

The World Heritage label does have some advantages in Africa, particularly where it can generate political interests at the national level. Regarding World Heritage and tourism in Africa, it is telling that many of Africa’s famous heritage sites, such as Ngorongoro, Kruger National Park, Great Zimbabwe, Zanzibar, Timbuktu, and the Serengeti National Park, are surrounded by a sea of poverty (Ndoro, 2015a). Here, the benefits of World Heritage inscription and the knock‐on effects generated by global tourism seem to be limited, and local communities are yet to see significant improvements in their lives and livelihoods.

There are also important issues to consider given the predominance of cultural sites focused on archaeology and European heritage in Africa’s World Heritage Site listings. Even the so-called “outstanding universal value” of Great Zimbabwe refers to the Queen of Sheba and the role of the site as a medieval capital. Here, World Heritage concepts and practices could learn from recent discussions arising from the *Black Lives Matter* movement about heritage and museum development. Most museums and heritage sites in Africa celebrate colonial history; rarely do we find museums dedicated to African achievements and liberation struggles (the exception being Angola with the promotion of liberation memorial sites and museums [Fig. 5]). The *Rhodes Must Fall* movement has clearly demonstrated the need to balance colonial history and African heritage (Chantiluke et al., 2018). Although some World Heritage experts may dismiss these movements as political machinations far removed from the realities of the archaeological science and architectural analysis, this risks giving credence to the idea that African heritage begins with the arrival of Europeans on the continent.

## UNESCO Gambian Heritage: Using a Site of Slavery to Build Capacity in Africa

Flordeliz T. Bugarin

Kunta Kinteh Island and Related Sites in The Gambia, West Africa, are prime examples of how the past can be used to address present concerns. This heritage zone was inscribed to the UNESCO World Heritage List in 2003 and includes seven sites: Kunta Kinteh Island, Six-Gun Battery, Fort Bullen, Ruins of San Domingo, Remains of Portuguese Chapel, CFAO Building, and Maurel Frères Building. From the fifteenth to twentieth centuries, the Kunta Kinteh Island and Related Sites landscape included interactions between Europeans and Africans in West Africa during the era of the Transatlantic Slave Trade and European colonialism. Located at the mouth of the Gambia River, it was a gateway to the interior of Africa. Europeans vied for control of the area by establishing forts and trading posts to enhance and control the trade of enslaved Africans and a variety of commodities. Given the significance of these sites in the history of this region, beyond the Senegambia and for people of African descent, it is paramount that appropriate conservation, restoration, and management strategies be strengthened. The enlistment of these sites as a UNESCO World Heritage Site is, therefore, appropriate in this regard. Since this site is meaningful for the global community, particularly people throughout Africa as well as the African Diaspora, the interpretation, conservation, and management of the site should include the voices and views of Black communities and consider their concerns. Engaging students of color and local African communities is a central part of preserving and understanding this site.

Since The Gambia is one of the poorest countries in Africa, it faces many challenges in terms of garnering the resources, finances, and technical capacity to fully conserve and properly manage its UNESCO World Heritage site and the related institutions. However, efforts have been made despite the many challenges, and international attention has been directed towards making improvements, including stabilizing the sites and collecting data that will lead towards better conservation strategies (Bugarin & Dunnavant, 2010; Bugarin, 2010a, b; Gijanto, 2009, 2013; Gijanto & Ceesey, 2018).

Kunta Kinteh Island and Related Sites have received 117,094 USD in international assistance through five approved requests between December 1996 and October 2016 (UNESCO, 2021). They have been granted these funds for a wide variety of purposes. This amount has covered the training of personnel for conservation work and preparation of a maintenance and monitoring plan. It has also aided in preparing the nomination of the site, and the conservation and partial restoration of Fort Bullen, a site subsumed under the Kunta Kinteh Island and Related Sites zone. As listed on the UNESCO World Heritage webpage (2021), funds were given in 2013 to review, update, and implement an integrated management plan for the site. In 2016, further funds were provided for the rehabilitation of the roof of the CFAO building in Albreda. Recently, the Director General of the Gambian National Centre for Arts and Culture (NCAC) has applied for funding from the USA Ambassador’s fund for cultural preservation to restore the island. In addition, the US Peace Corps has occasionally supported conservation initiatives through the efforts of individual volunteers. The impacts of these collective efforts have led to some critical improvements, but more still needs to be done to fully mitigate rapid decay of the structures and landscape. In addition, more funds could be garnered to fully include members of the local communities in conservation efforts and to strengthen their capacity to manage the site and create related businesses that improve their standard of living.

UNESCO World Heritage status helps archaeologists to recruit volunteers and local community members for conservation and archaeological projects, while also providing opportunities for heritage experts and managers of archaeological sites to train a wide range of stakeholders. Participating in an excavation at a UNESCO World Heritage site is particularly attractive to many students. This experience may inspire them to return to The Gambia or other sites throughout Africa and the African diaspora.

**Fig. 6:**
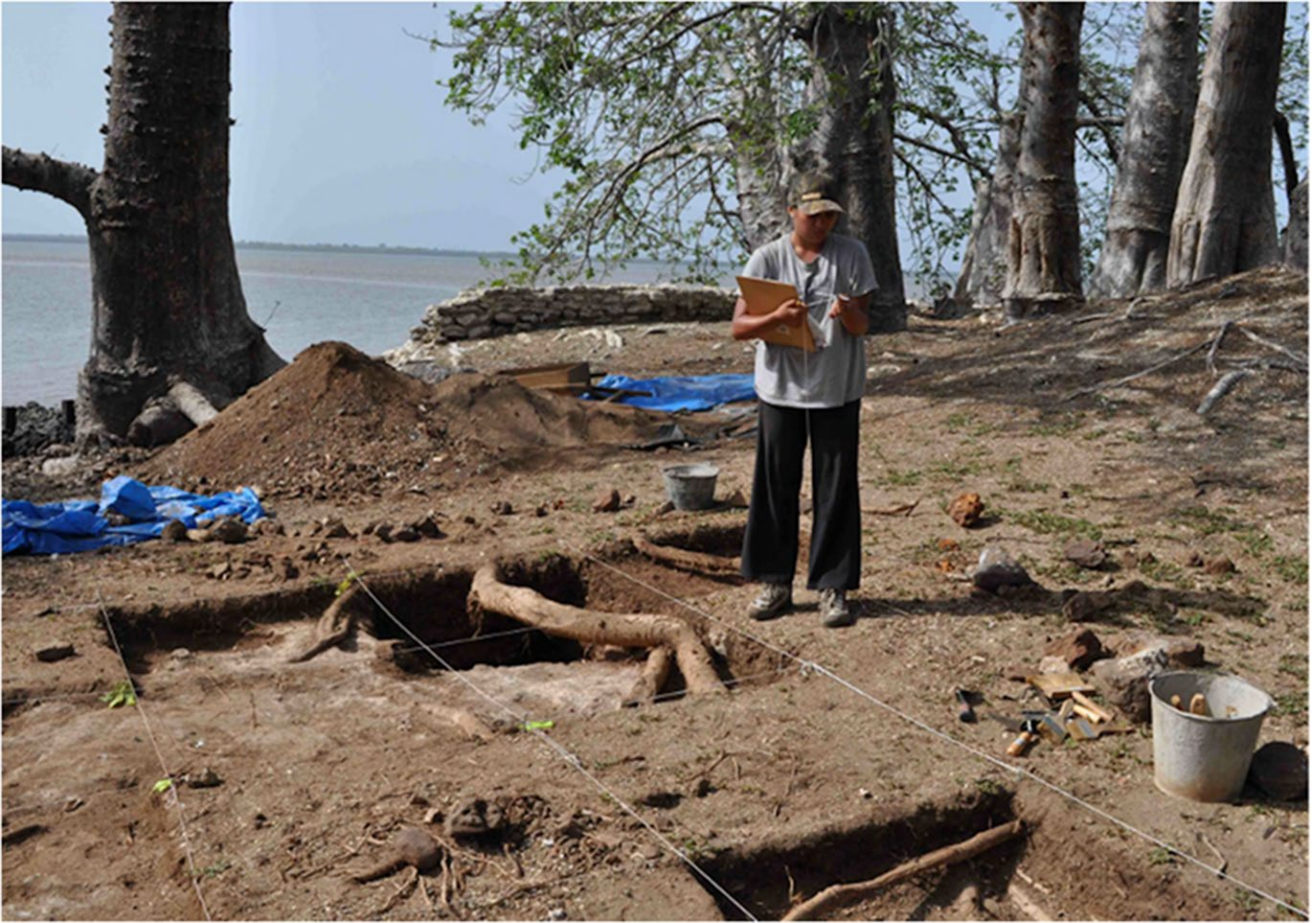
Howard University student Katrina Aben excavating on Kunta Kinteh Island, The Gambia (photo by F. Bugarin)

For example, students under my supervision from Howard University have been able to work side by side with Gambian community members on archaeological excavations investigating the activity areas left by enslaved people who lived on Kunta Kinteh Island (Fig. 6). They also took turns giving presentations on our research to daily visitors, a process that helped them internalize and express the history of the site, archaeological theory and methods, and interpretive perspectives of daily findings. Collections were brought back to Washington, D.C., and more Black students were able to work with the artifacts. For students at Howard University, and other historically black colleges and universities (HBCUs) in the USA, these experiences put the Black material past directly in their hands. They enable African American students to engage in the archaeological and historical reconstruction, documentation, preservation, writing, and interpretation of the Black diasporic past, while also giving them a sensory connection to Africa and a global Black past. Including African American students in the stewardship of sites and inspiring students of color to be emotionally invested in these sites are paths towards ensuring the sustainability of these heritage resources.

**Fig. 7:**
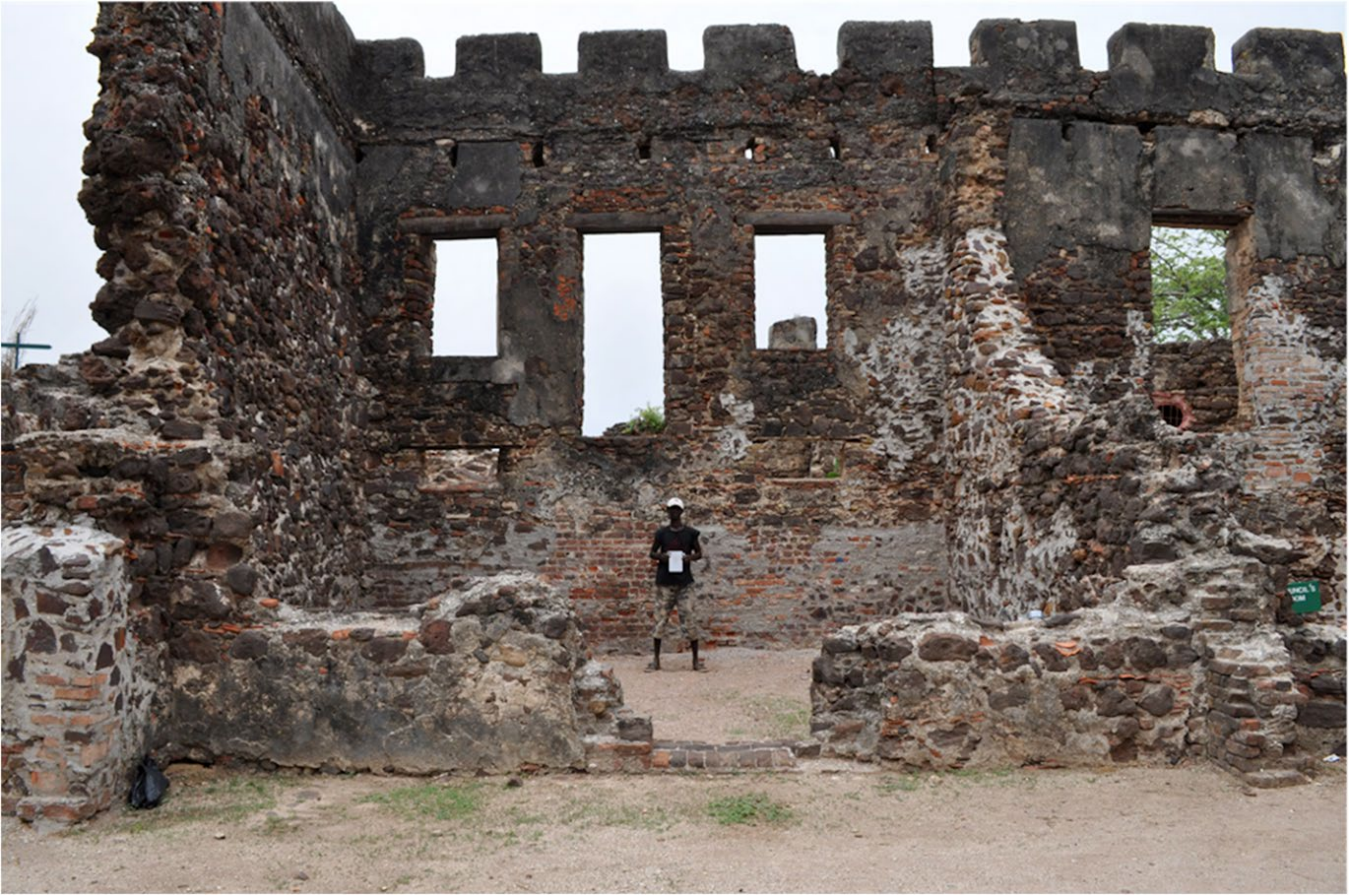
Local community member Aboulie Jabang standing in the ruins of Fort James, The Gambia (photo by F. Bugarin)

Sites that interest HBCU students, and others throughout the diaspora, have become part of the agenda for transforming archaeology and heritage studies and enhancing human capacity in Africa. For students of color at Howard University and elsewhere, experiential learning serves to insert them in the scientific and humanistic process of archaeology and the sociopolitical process of building capacity and uplifting contemporary Black communities. For the local African crew members who worked with us (Fig. 7), most of whom had a basic level of education, the project provided an opportunity to engage in peer-to-peer learning and improve their basic reading and writing skills.

Howard University students and community members were also included in the interpretation process on Kunta Kinteh Island. Field and lab work were embedded in an experiential learning process that encouraged everyone to share their perspectives on the past. The students were given the opportunity to discuss and document their thoughts about how they experience the site, internalize the reconstructed past, and feel about holding objects that belonged to enslaved people. The experience gave them a more intimate and sensorial look at this past in comparison to what they learn from textbooks and classroom lectures. Furthermore, sharing this experience presented US-based Black students, other students of color, and Gambians the opportunity to build friendships, as critical bridges between Africa, the USA, and others in the diaspora.

The Du Boisian approach to education calls upon the “Guiding Hundredth” to instill radical change through group leadership (DuBois, 1973). Thus, it is important to bring Howard University students to the table to not only learn archaeology, but also to internalize the experience of documenting and explaining the past of enslaved peoples and to live the modern challenges facing local African communities. Requiring students to explain our goals and the history of the site to tourists builds leadership skills and enables them to reflect upon and articulate their relationship to the site. Several faculty members at Howard University have recognized the potential benefits from a Du Boisian approach that lays the seeds for a life-long, service-oriented commitment between Black students and learning communities throughout Africa and the African diaspora (Verharen et al., 2017), but this requires Africana universities to push for radical change, particularly in terms of science, poverty alleviation, and capacity building. As Howard students worked on Kunta Kinteh Island, they were able to interact with visiting tourists, particularly Black tourists, who expressed appreciation for seeing students of color and an HBCU taking an interest in preserving and documenting a Black past. The engagement of Black diasporic students and institutions in the preservation of UNESCO World Heritage Sites housed in Africa and devoted to Black history is particularly salient, especially if stakeholders seek to build bridges between Africans and their diasporas.

In addition to impacting the general global community, these types of sites have the potential to influence our larger body of work. Capturing African and diasporic voices in the study and interpretation of archaeological and heritage sites can make important contributions to the decolonization of the professions and scholarship of archaeology, history, cultural studies, and conservation. The call for this decolonizing action has long been in the making and is increasingly being echoed throughout the world.

## UNESCO World Heritage List and the Sahara, Seen from SW Libya

Savino di Lernia

**Fig. 8:**
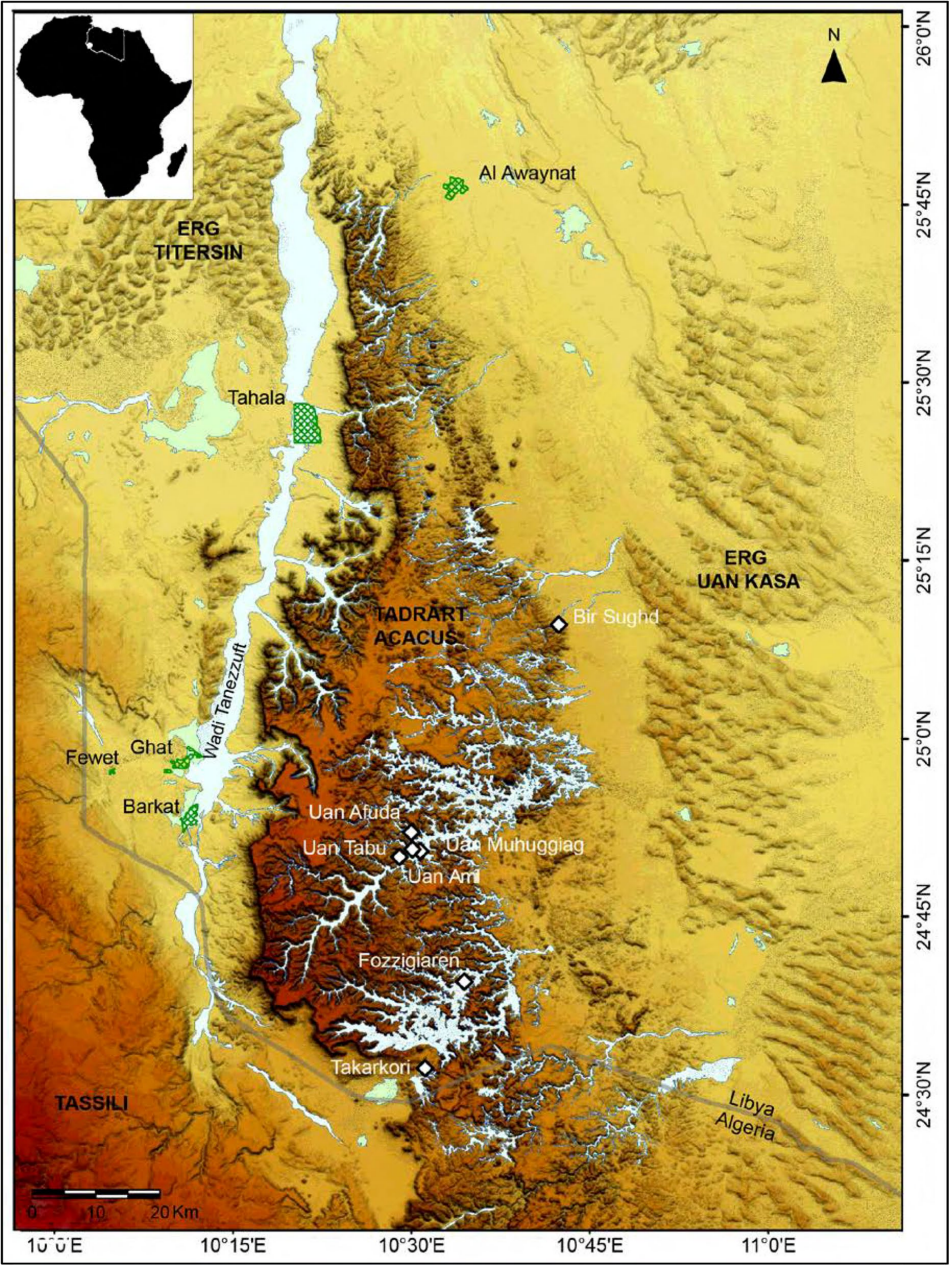
The Tadrart Acacus massif, Libya (after Gallinaro 2013)

In a recent *African Archaeological Review* paper (di Lernia, 2018), I commented on some of the problems related to the (very few) Saharan properties included in the UNESCO World Heritage List. Although the peculiar division between properties listed as “African” vs. those falling under the rubric “Arab states” creates some problems in the analysis of numbers, these nevertheless remain very low. In addition, there is no reference to the Sahara as a classificatory “container.” Here, I will discuss some contexts that broadly fall within today’s Sahara Desert, which does not include sites along the Nile Valley (i.e., Memphis, the Pyramid Fields, Ancient Thebes, and the Nubian Monuments from Abu Simbel to Philae). More specifically, I will discuss the Tadrart Acacus property in SW Libya (Fig. 8), where I worked uninterruptedly from 1990 until a few years after the “Arab Spring.”

Today, ten UNESCO World Heritage properties—cultural, natural, and mixed (Table 1)—could be theoretically related to the Sahara. Following the ratification of the World Heritage Convention in 1972, it took ten years to have the first two Saharan sites inscribed, which were both in Algeria (M’Zav valley and Tassili n’Ajjer). Nearly 50 years after the Convention (1972–2020), we could safely argue that the inscription of a Saharan property in the World Heritage List, or even the Tentative List, is a rather episodical event. The main causes for such low numbers are probably economic crisis, political instability, and ongoing turmoil. Based on my Libyan experience, however, the inscription of the Tadrart Acacus on the World Heritage List did not affect its political management, either before or after the Libyan conflict.

**Table 1:** “Saharan” properties inscribed in the UNESCO World Heritage List (source: https://whc.unesco.org/en/list/, accessed February 16, 2020)

State	Name of the property	Type	Date of inscription	Criteria	Size (ha)	Buffer zone (ha)	In danger
Algeria	*Ksour* of the M'Zab Valley	Cultural	1982	(ii), (iii), (v)	665.03	n/a	–
Algeria	Tassili n'Ajjer	Mixed	1982	(i), (iii), (vii), (viii)	7,200,000	n/a	–
Chad	Lakes of Ounianga	Natural	2012	(vii)	62,808	4869	–
Chad	Ennedi Massif: Natural and Cultural Landscape	Mixed	2016	(iii), (vii), (ix)	2,441,200	777,800	–
Libya	Rock-Art Sites of Tadrart Acacus	Cultural	1985	(iii)	3,923,961	n/a	Since 2016
Libya	Old Town of Ghadamès	Cultural	1986	(v)	38.4	n/a	Since 2016
Mauritania	Ancient *Ksour* of Ouadane, Chinguetti, Tichitt and Oualata	Cultural	1996	(iii), (iv), (v)	n/a	n/a	–
Mauritania	Banc d'Arguin National Park	Natural	1989	(ix), (x)	1,200,000	n/a	–
Niger	Air and Ténéré Natural Reserves	Natural	1991	(vii), (ix), (x)	7,736,000	n/a	Since 1992
Niger	Historic Centre of Agadez	Cultural	2013	(ii), (iii)	77.6	98.1	–

**Fig. 9:**
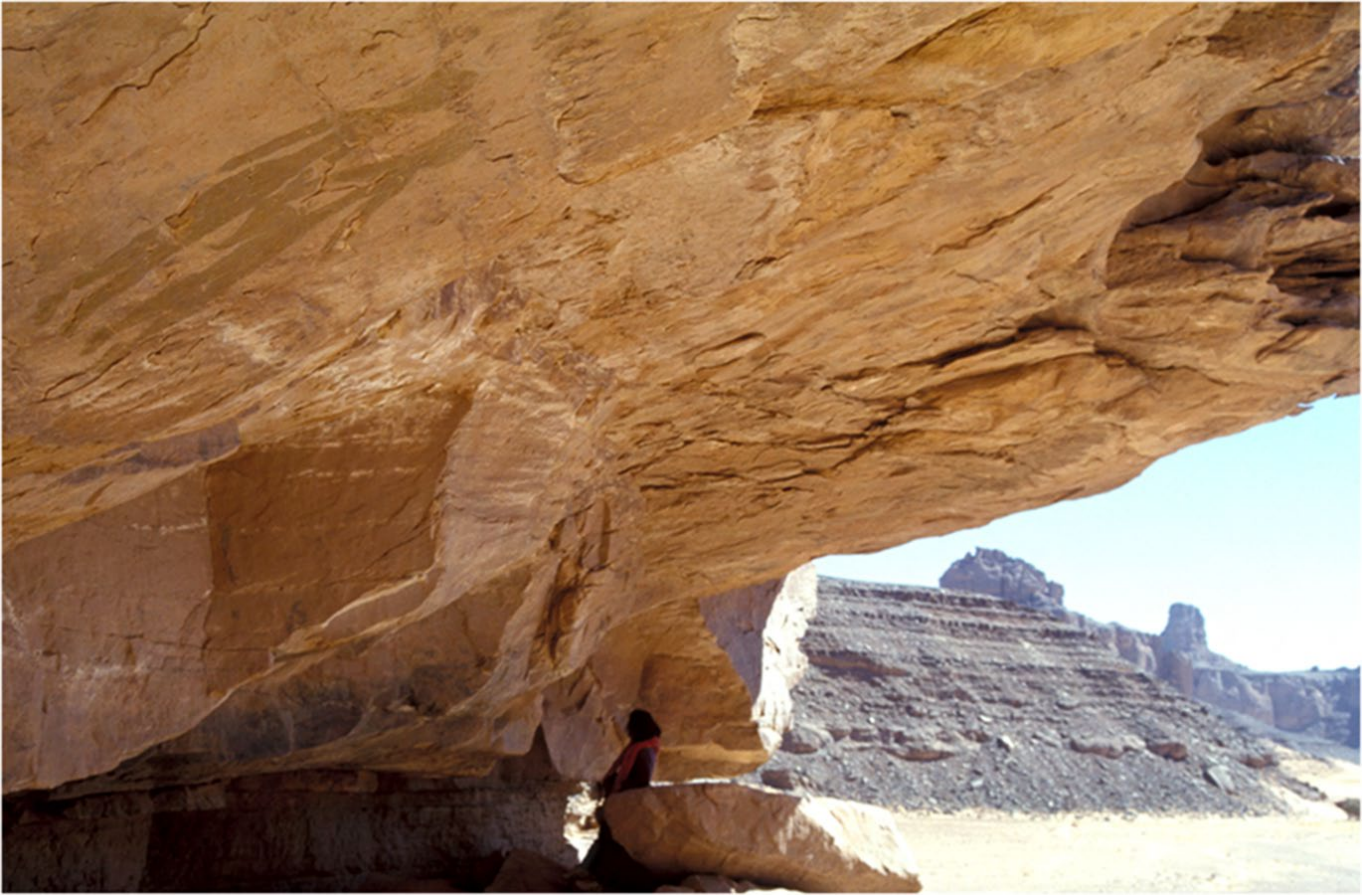
Round Heads anthropomorphs from Afozzigiar, a rock shelter in southern Acacus (photo courtesy of the Archive of the Archaeological Mission in the Sahara, Sapienza University of Rome)

Between 1999 (lifting of UN embargo) and 2011 (beginning of the “revolution”), the Tadrart Acacus massif—inscribed in the list for its extraordinary rock art (Fig. 9)—was a top tourist destination, with approximately 100,000 visitors per year, mainly between October and March. However, despite the site being advertised for its world-renowned rock art, only one in ten tourists came for this reason; the landscape and the presence of the Tuareg were the true motivation for most visits (Sergio Scarpa, pers. communication). In contrast to other top tourist destinations around the world, the microeconomy of the Ghat region and Germa (ancient *Garama*, capital of the Garamantian kingdom) largely benefitted from this tourist flux. Most cars, drivers, and guides were recruited locally. Food and accommodation were also “local.” Unfortunately, Libyan institutions never took the necessary steps to create a national park in the region, despite several calls to do so (e.g., Liverani et al., 2000). Local agencies simply collected the fee of a few Libyan Dinars (less than 3 Euro) charged to each car entering the Tadrart Acacus. In some cases, the tickets were sold by the local office of the Department of Antiquities in Ghat, but information about the official amount of the entrance fees and the ways this money was used is not available (to me).

One of the benefits of placing Tadrart Acacus on the UNESCO World Heritage List was protection from illegal construction and oil exploration. Between the mid-1980s and the early 2000s, the area was largely affected by oil exploration activity, including the creation of seismic lines in the nearby Messak plateau and the sand seas of the Erg Uan Kasa and Edeyen of Murzuq. The scale of the damage was large enough to attract international attention (Kröpelin, 2002), which eventually led to compensation actions: surveys and risk assessments were in fact financially supported by the interested oil companies, supervised by the Libyan Department of Antiquities (Anag et al., 2002). Yet, the protection of the Tadrart Acacus might be considered, in a sense, at the expense of other places, such as the Messak Settafet and the Messak Mellet, where damage is irreparable. This leads us back to the role of state institutions and other stakeholders involved in the nomination process: Had the Messak been included in the UNESCO World Heritage List in the early 1980s, would it have suffered the same damage? And why was it not included in the first place? It is based on this unfortunate experience in Libya that I have called for the reinsertion of Niola Doa, in the Ennedi Massif in Chad, as a proper UNESCO World Heritage property and not simply a buffer zone as it stands now (and where oil exploration is planned), for which management rules are rather different (see di Lernia, 2018).

Furthermore, we should not forget that the people living in these sites should have a say on this matter. The Tadrart Acacus is home to one of the few Tuareg groups (the kel Tadrart) who still live in these mountains, use traditional dwelling structures (locally called *zeriba*), and practice goat herding and occasionally rainfed cultivation (di Lernia et al., 2012). These communities are rarely included in the conversation on heritage matters. In 2009, episodical but dramatically effective vandalism of several rock art paintings in central and northern Acacus (di Lernia et al., 2010; see also Chalcraft, 2014) led the Minister of Tourism to call for a total closure of the region to everyone, including to the kel Tadrart! The role of the kel Tadrart as “custodians” of the Tadrart Acacus has been requested and invoked many times (Bennett & Barker, 2011), but never actually promoted. Most of the people working in the area during the “peak tourist years” were from Mali or Niger, mostly because they could speak English or French, unlike the locals, who were limited by the constraints of the Ghaddafi regime, where other languages were scarcely practiced and substantially hindered. For example, there was an almost total absence of bilingual signs in public places, including airports and along the main roads, until the early 2000s. The difficulty of defining a buffer zone also has important implications for the people living in and around the property: several monumental contexts are at the base of the mountain range, along the Wadi Tanezzuft valley, west of the Tadrart Acacus, and to the east, along Wadi Imessarejan and Wadi Awiss. These are close to reclamation areas, infrastructure, and motorways. No management plan is active, and the risk is high. For example, several burial grounds have been heavily damaged in the last few years due to use of bulldozers to remove stones for new construction projects—sometimes illegally because they lack the necessary permits (Ali Khalfalla, pers. comm.). If the buffer zone had been officially defined, and the rules of use clearly indicated by a legislative act, perhaps some of these sites would have fared better. Overall, the importance of defining a buffer zone is most keenly felt for those properties defined and included in the World Heritage List long ago—such as the Tadrart Acacus in Libya—where the property boundaries have remained poorly defined until recently, with critical consequences for the cultural heritage of this area.

At a different scale, the inscription of the Tadrart Acacus in the UNESCO World Heritage List was essential for its protection, especially in the context of the ongoing Libyan conflict. During the first phase of the conflict (approximately March–November 2011), the Acacus was one of the first sites inserted in the NATO (North Atlantic Treaty Organization) no-strike list, together with the other Libyan UNESCO sites and a few other locations. Given the deterioration of the situation in Libya (di Lernia, 2015), which is still worsening, the Tadrart Acacus and four other UNESCO World Heritage sites (Leptis Magna, Sabratha, Cyrene, Ghadames) have been placed on the List of World Heritage in Danger. Unfortunately, no independent official mission has been carried out in southern Libya since 2013, so it is difficult to get first-hand information.

To conclude, despite some gray area, the experience of the Tadrart Acacus as a World Heritage Site seems to be positive rather than negative. A step to be taken in the immediate future, however, should be towards a redefinition of this property as a “cultural landscape.” According to UNESCO, cultural landscapes “represent the combined works of nature and of man. They are illustrative of the evolution of human society and settlement over time. Protection of cultural landscapes can contribute to modern techniques of sustainable land use and can maintain or enhance natural values in the landscape. The continued existence of traditional forms of land use supports biological diversity in many regions of the world. The protection of traditional cultural landscapes is therefore helpful in maintaining biological diversity” (UNESCO, 2020). Based on its unique blend of cultural and natural features, the Tadrart Acacus no doubt deserves such a new designation.

## The Local Community and Tourism at Kilwa Kisiwani World Heritage Site, Tanzania

Elgidius B. Ichumbaki & Noel B. Lwoga

### Introduction

As part of a program to popularize Tanzania’s cultural heritage, one of us (EI) and a team of journalists visited Kilwa Kisiwani (KK) in 2016 to produce radio and television programs. These media outputs aimed to inform the public about the natural beauty of KK Island and its important cultural heritage. To accomplish these aims, the team engaged with local people to capture their voices. One of the dilemmas that the team encountered was the reaction when local people were asked to talk about benefits they gain as a result of living within a World Heritage Site.

One of the respondents was *Bibi*, a woman of 70 + years sitting in her house’s exterior lobby (*Bibi* is a Swahili word respectfully signifying an “elderly woman”). When asked how she feels to live in a World Heritage Site, she remained quiet for 2 min or 3 min. She then struggled to move further away from the lobby and stood where she could easily view Kilwa Masoko, a township on the mainland where the Antiquities office is located and where visitors to KK pay their entrance fee before they catch a boat coming to the island. Pointing her finger at the mainland, she narrated: “*Kisiwani na magofu yake vinaliwa pale; wanaovifaidi wako pale. Kisiwani huwa wanakuja wageni tofauti tofauti. Leo wanakuja wageni hawa, kesho wanakuja wageni wale. Sisi tunawaona tu wanazunguka zunguka na kuwanufaisha wa pale; Sisi wa Kisiwani tunaishia kutimuliwa vumbi tu*.” Translation: “The Island of KK and its ruins do not benefit residents. The beneficiaries are on the mainland. We [residents of KK] do not benefit. We observe tourists visiting the ruins on daily basis and their traffic creates a significant amount of dust.” This narrative regarding the benefits from tourism broadly captures what KK residents know about living in a World Heritage Site. Building on this scenario, in this short article, we present the extent to which the local community in KK has either gained or lost from living in the vicinity of a World Heritage Site.

### Kilwa Kisiwani: a Short Description

**Fig. 10:**
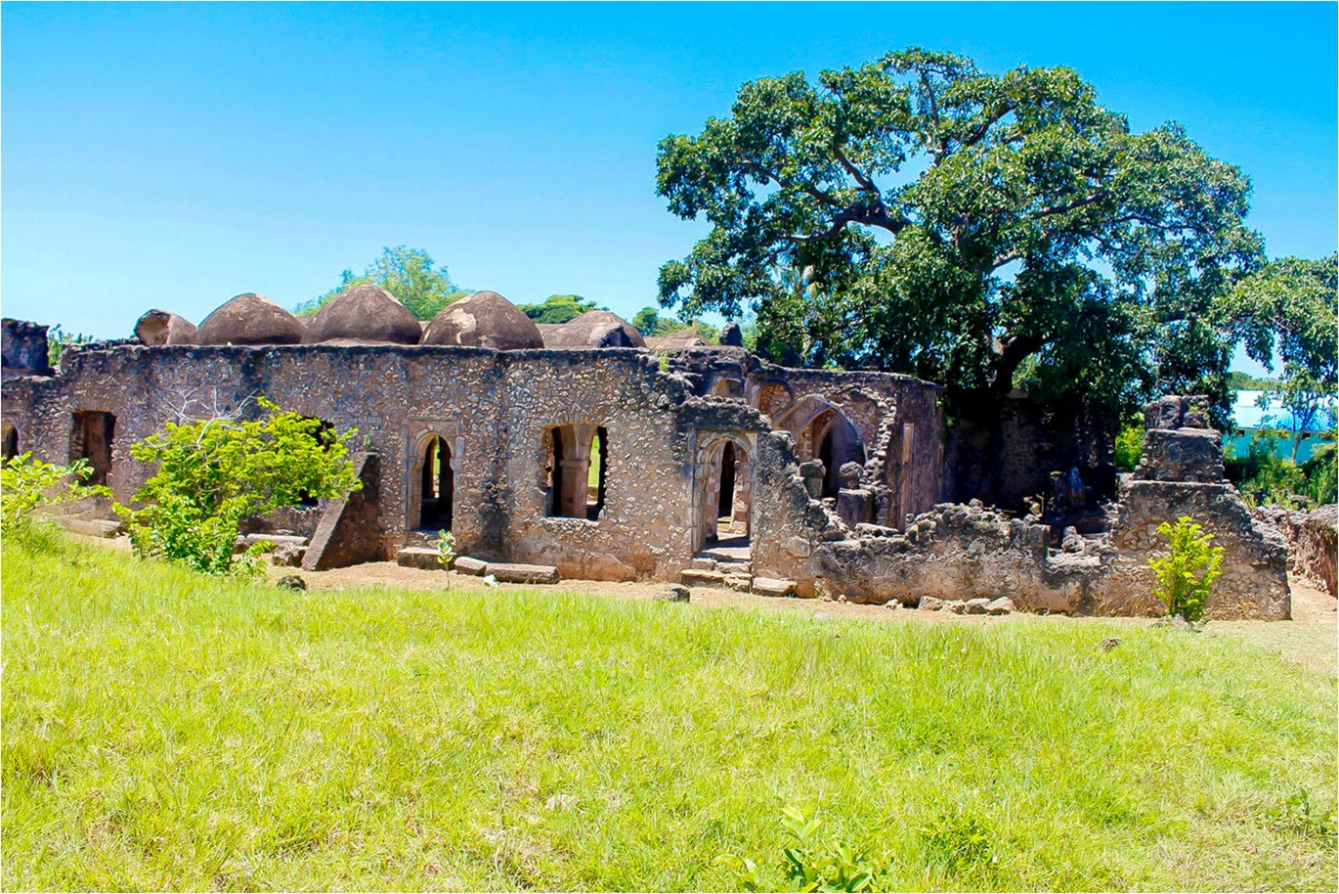
The Great Mosque of Kilwa Kisiwani, Tanzania (photo by E. Ichumbaki)

The ruins of KK are a part of UNESCO’s World Heritage Sites along the southern coast of Tanzania. In the late 1930s, the British colonial government in Tanganyika (now Tanzania Mainland) declared these ruins national monuments based on the provisions of the Monument Preservation Ordinance of 1937 (Ichumbaki, 2016). After independence in 1964, the ruins were protected under the Antiquities Act of 1964, which was amended in 1979. Later, UNESCO inscribed the KK ruins together with those of a nearby island of Songo Mnara on the World Heritage List in 1981. Together, these islands provide exceptional architectural, archaeological, and documentary evidence for the growth of Swahili culture and commerce along the East African coast from the ninth to the nineteenth centuries. The ruins of KK represent visible and tangible evidence of Swahili civilization and bear unique testimony to the expansion of Swahili coastal culture. The Great Mosques (Fig. 10), for example, signify the introduction of Islam to the region during the late first millennium AD. Abundant cultural objects, such as imported ceramics and glass beads, indicate the prosperity of KK during the medieval period. The authenticity and integrity of KK offer important insights into the economic, social, and political dynamics of the East African coast and its mainland.

The island of KK has a rich maritime history involving transoceanic links with Asia and Europe. During the late medieval period, KK controlled gold trade from the port of Sofala in modern Mozambique and dominated a large part of the Swahili coast. This history is evident in the ruins of stone monuments, including palaces, mosques, and forts connected to the sea by ports and landing places still in use today. The local people in KK continue their rich maritime connections through traditional dhow building, fishing, shell gathering, seafaring skills, and oral traditions about shipwrecks, slaves, and giants (Chittick, 1974, p. 414; Pollard et al., 2016, p. 358). In KK, there are over 1,200 people today whose livelihoods depend on Indian Ocean resources (Ichumbaki & Mapunda, 2017; Ichumbaki & Pollard, 2020).

### Tourism Potentials in KK

**Fig. 11:**
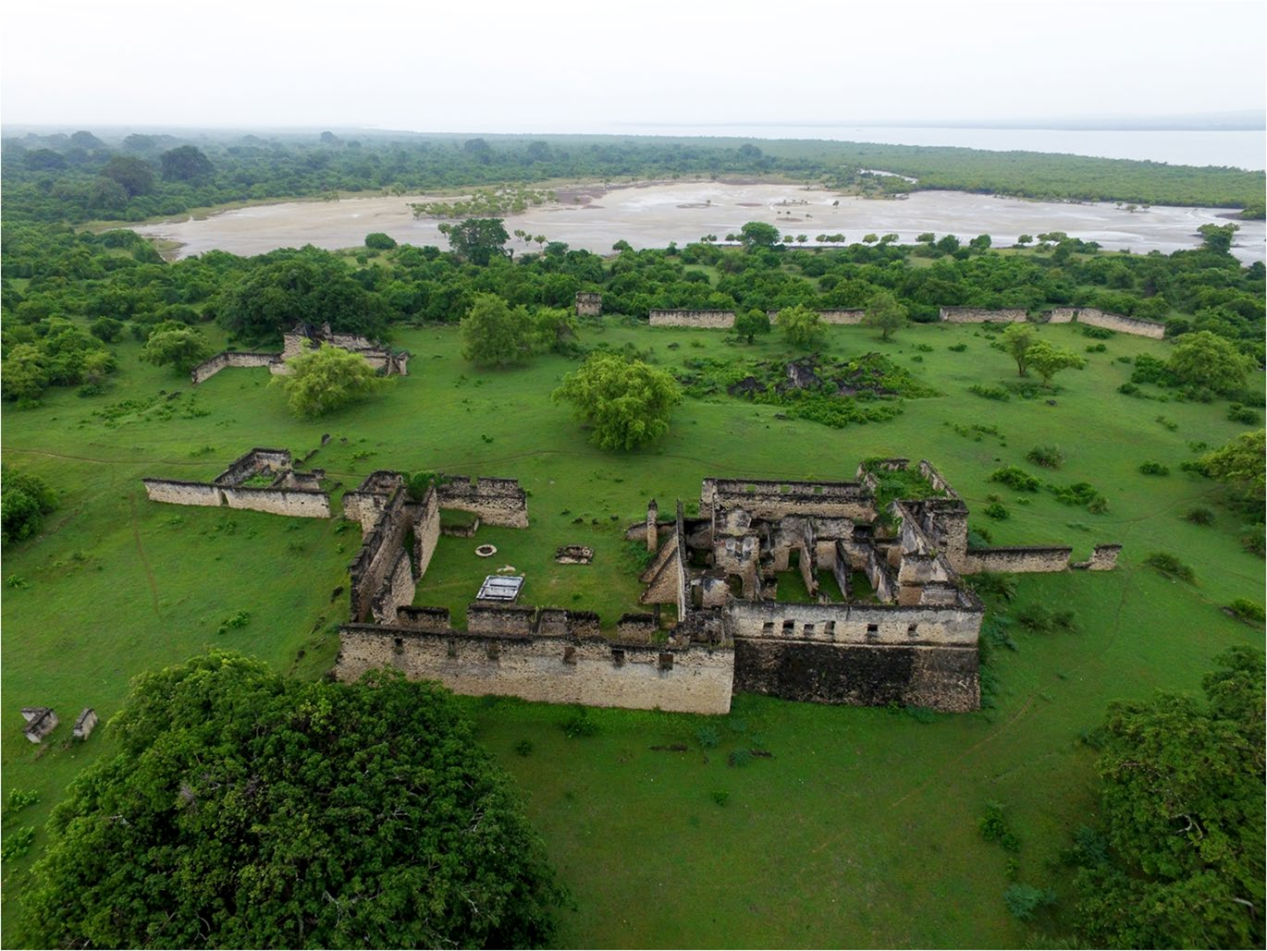
Aerial view of the Makutani Palace in Kilwa Kisiwani, Tanzania (photo by E. Ichumbaki)

In terms of tourism potential, KK has lots to offer. There are two domed mosques—the small and great mosques—that testify to the history of the Islamic faith on the Island. There are also the remains of monumental buildings such as Husuni Kubwa and Makutani Palace. Whereas Husuni Kubwa signifies the hegemony of KK in controlling the gold trade during the fourteenth century, Makutani Palace (Fig. 11) is a testimony to the revival of KK’s power and influence in the eighteenth century. Other monumental structures that usually capture the attention of tourists in KK include an Old Fort overlooking the sea, stone tombs of various shapes scattered across the Island, and medieval wells that sustained the local population. Tourism potentials that remain underexplored in KK include the various material culture including collections of Kilwa coins, local and imported ceramics and beads of different types, exceptional navigational complexes such as causeways, and beautiful beaches.

Additionally, there are shipwreck sites dating from the eighth to the fourteenth centuries. The first one is around *Jiwe la Jahazi* (“stone of the dhow”), a 3-m high islet on a 38-m long reef crest which looks like it has the bow and stern of a boat from a distance (Pollard et al., 2016, pp. 357–359). Intertidal surveys conducted around *Jiwe la jahazi* have recorded a number of basalt blocks, large sandstone cobbles and boulders, and a number of ceramics imported from the Persian Gulf. These materials suggest that a vessel carrying imported goods from the Gulf wrecked at *Jiwe la Jahazi* between the eighth and tenth centuries (Pollard et al., 2016, p. 360). The second shipwreck site is off the KK port. Geophysical and underwater surveys up to 10 m deep revealed an artifact dispersion on the seabed around 100 m from the low watermark. Cultural materials recorded on the seabed included a medieval stone anchor, local ceramics, and pottery imported from southwest Asia (Pollard et al., 2016, p. 360). This underwater site, which features large numbers of artifacts, such as pots, bones, shells, angular limestone, quartz, sandstone, and basalt, has not been opened to tourists.

### Beneficiaries of Tourism in KK

KK is part of the Southern Tourism Circuit, which is currently the focus of Tanzania’s tourism development plans. The island received about 3,048 tourists in 2018, making it fifth among cultural heritage sites in the country in terms of tourist visits (URT, 2019). However, people living in KK do not accrue economic, sociocultural, and environmental tourism benefits. They are neither employed in tourism services, nor do they earn income from selling local products. The local communities miss both employment and entrepreneurial skills because there have not been deliberate, inclusive interventions to enable them to benefit from heritage in the region. The beneficiaries of tourism are the Tanzania Wildlife Authority (TAWA), which charges entrance fees, and non-local tourism operators (transporters and hoteliers), who provide transport and accommodation services. The latter are neither from Kilwa, nor do they have offices there, and they hire tour guides from Kilwa Masoko township.

The local guides of KK have a little market share, including domestic student groups and some backpacker tourists who enjoy individual travel coordination. Members of the local KK community would like to earn income by selling boat transportation, shop products, and local crafts. Unfortunately, they cannot achieve these goals due to their lack of experience, entrepreneurial skills, and capital. The community members would also like to receive dividends from government collections, including tourist entry fees and the taxes and licenses that the government charges tour operators and accommodation facilities (Lwoga, 2018). Unfortunately, nothing is forthcoming. Although the government and other tourist agencies do not bar people in KK from establishing and investing in tourism businesses, there are no policies and procedures that would help them to engage the informal tourism sector. Before the World Heritage listing, local community members were free to conduct various activities, such as farming and raising of domestic animals, but now they are no longer allowed to build houses, or even dig toilet holes, without consulting the Antiquities Department and other government authorities mandated to care for the monuments. Generally speaking, the lives of KK people have been downgraded in relation to monument preservation activities and a flourishing tourism industry that fails to give back to the community. The Tanzanian government claims to give back by funding public services and other facilities, but this is not the case because the social services in Kilwa Kisiwani are poor. There is a lack of safe drinking water, health facilities, and a well-furnished primary school, and the absence of a ferry means there is no reliable transportation (Ichumbaki & Lubao, 2020, p. 424).

### Some Suggestions

The island of KK has exceptional tourism potential, but it remains undeveloped in ways that could attract tourists and generate income for the local community. Further developments to support tourism include setting up lodges and guest house accommodations on the island and expanding the current site museum to exhibit unique objects such as the Kilwa coins, various glass and non-glass beads, and ceramics and other cultural objects that are important to the history of KK. Another important move would be to support local artisan groups who could make materials to sell to tourists. These materials would include clothing, beads, pottery, and other souvenirs that are based on the history of KK and maritime cultural heritage of the area. Within the environs of the recently established site museum, there could be a summary of the site history and demonstrations of local crafts, such as boat building. This would contribute to creating a sustainable and healthier local community on Kilwa Island.

These local initiatives may seem difficult, but initial trials supported by the University of Dar es Salaam and Escala Initiatives, an American non-profit enterprise, seem to work well. This project has enabled a group of 20 women on the island to develop their entrepreneurial skills, leading to short-term benefits from working with research teams to prepare meals and collaborate on ethnographic research. With this training, these women have also been able to invest in making cultural objects such as baskets which they now sell to tourists. Furthermore, the women have been able to produce inexpensive soap for sale in the local community, helping to promote handwashing as a highly recommended measure during COVID-19 pandemic. With the arrival of tourists who can stay longer on the island, these women will have more opportunities for other businesses in the long term. There is also a need to develop more trails to sites around the Island, thus encouraging tourists to stay longer and invest more in the local community by hiring local guides and buying food and accommodation. An implementation of these suggestions, together with many others offered elsewhere (see Ichumbaki & Mapunda, 2017), could result in a more productive use of ruins to benefit the local population in KK.
